# Melatonin in plant pathogen defense: a review of its role in horticultural crops

**DOI:** 10.1093/hr/uhaf150

**Published:** 2025-06-11

**Authors:** Xinyi Hao, Jinyu Ren, Mingyuan Xu, Binghui Sun, Rui Li, Shijin Yang, Weirong Xu

**Affiliations:** School of Enology & Horticulture, Ningxia University, Yinchuan 750021, Ningxia, China; Engineering Research Center of Grape and Wine, Ministry of Education, Ningxia University, Yinchuan 750021, Ningxia, China; Ningxia Engineering and Technology Research Center of Grape and Wine, Ningxia University, Yinchuan 750021, China; State Key Laboratory of Efficient Production of Forest Resources, Beijing Forestry University, Beijing 100080, China; School of Enology & Horticulture, Ningxia University, Yinchuan 750021, Ningxia, China; Engineering Research Center of Grape and Wine, Ministry of Education, Ningxia University, Yinchuan 750021, Ningxia, China; Ningxia Engineering and Technology Research Center of Grape and Wine, Ningxia University, Yinchuan 750021, China; School of Enology & Horticulture, Ningxia University, Yinchuan 750021, Ningxia, China; Engineering Research Center of Grape and Wine, Ministry of Education, Ningxia University, Yinchuan 750021, Ningxia, China; Ningxia Engineering and Technology Research Center of Grape and Wine, Ningxia University, Yinchuan 750021, China; School of Enology & Horticulture, Ningxia University, Yinchuan 750021, Ningxia, China; Engineering Research Center of Grape and Wine, Ministry of Education, Ningxia University, Yinchuan 750021, Ningxia, China; Ningxia Engineering and Technology Research Center of Grape and Wine, Ningxia University, Yinchuan 750021, China; School of Enology & Horticulture, Ningxia University, Yinchuan 750021, Ningxia, China; Engineering Research Center of Grape and Wine, Ministry of Education, Ningxia University, Yinchuan 750021, Ningxia, China; Ningxia Engineering and Technology Research Center of Grape and Wine, Ningxia University, Yinchuan 750021, China; School of Enology & Horticulture, Ningxia University, Yinchuan 750021, Ningxia, China; Engineering Research Center of Grape and Wine, Ministry of Education, Ningxia University, Yinchuan 750021, Ningxia, China; Ningxia Engineering and Technology Research Center of Grape and Wine, Ningxia University, Yinchuan 750021, China; School of Enology & Horticulture, Ningxia University, Yinchuan 750021, Ningxia, China; Engineering Research Center of Grape and Wine, Ministry of Education, Ningxia University, Yinchuan 750021, Ningxia, China; Ningxia Engineering and Technology Research Center of Grape and Wine, Ningxia University, Yinchuan 750021, China; State Key Laboratory of Efficient Production of Forest Resources, Beijing Forestry University, Beijing 100080, China

## Abstract

Horticultural crops have important economic value in the world. Biotic stress has serious impacts on horticultural crops’ growth and development as well as yield. Melatonin, a multifunctional signaling molecule, has been increasingly documented to play a pivotal role in mediating plant defense responses against diverse biotic stressors, including bacterial, fungal, and viral pathogens in horticultural crop species. Previous studies showed that exogenous melatonin treatment significantly improved horticultural crops growth and increased their tolerance to biotic stress. Although there are numerous studies to show that exogenous melatonin treatment can markedly improve the tolerance for horticultural crops in response to biotic stress, the role of melatonin in biotic stress responses remains unclear and requires clarification. In the review, we summarize the effects of melatonin on horticultural crops’ disease resistance. Moreover, we assess future perspectives in melatonin research and its applications to improve horticultural crop production and tolerance for biotic stress. This review explores future research directions and potential applications to enhance the productivity and biotic stress tolerance of horticultural crops, and also provides a theoretical basis for enhancing the scientific understanding of the role of melatonin in response to biotic stress in horticultural crops.

## Introduction

Horticultural crops play an important role in our life and are a major source of minerals, micronutrients, proteins in human nutrition [1, 2]. Especially in developing countries, it is also a key source of income generation and poverty alleviation [3]. With the rapid development of the world economy, people have an increasing demand for high-quality horticultural crops. However, bacteria, fungi, viruses, and others biotic stresses seriously threaten the development and growth of horticultural crops, resulting in quality and yield loss [4–9]. Melatonin was first identified from the bovine pineal gland [10], and the first application to be a plant growth regulator was in 1995 in plant [11, 12]. Previous evidence has demonstrated that melatonin performs a plethora of functions in horticultural crops in mitigating biotic stress [13–15]. Recently, many studies have reported that melatonin also plays a vital role for horticultural crops in response to biotic stress by inhibiting cell growth, fungicide susceptibility, virus replication, and homeostasis of gene expression [16–28].

With the potential functions of melatonin excavated in response to the biotic stress in horticultural crops, exogenous melatonin is widely applied in the preservation of horticultural crops from biotic stress damage and improves horticultural crops’ stress tolerance, thus increasing production [16, 18, 19, 26–28]. In addition, it further draws focus on exploring the potential benefit in the horticultural system for breeders and pathologists of horticultural science. Hence, a comprehensive review is necessary to summarize the melatonin’ involvement in the physiological and molecular responses in horticultural crops in response to biotic stress. Here, we systematically summarize the research on melatonin application in horticultural crops, and also focus on the future directions as well as its applications for improving horticultural crops in response to biotic stress.

## Melatonin for alleviating pathogen-induced diseases

Melatonin plays a significant role in horticultural crops’ responses to biotic stress. In this section, recent studies on melatonin involved in responses to biotic stress will be summarized.

## Bacterial diseases

Bacteria stress, is as one of most, common biotic stresses that seriously affects the horticultural crops growth, resulting in huge yield loss, and recent evidence shows that melatonin is beneficial in dealing with bacteria stress in horticultural crops [20, 29–33]. For example, cassava bacterial blight, a serious disease in *cassava*, has been effectively alleviated via regulation of melatonin biosynthesis genes, and further study found that *MeRAV1* and *MeRAV2* were upregulated, playing a role in melatonin biosynthesis and improved the endogenous melatonin through virus-induced gene silencing in cassava leaves. Furthermore, the genes related to melatonin biosynthesis in cassava can also positively regulate plant disease resistance [20]. In *Citrus*, Huanglongbing associated to *Candidatus Liberibacter asiaticus* (CLas) is a devastating citrus disease worldwide and melatonin also plays a critical role in defending against Huanglongbing *in citrus*. Further study showed that Valencia sweet orange infected by Huanglongbing markedly increased endogenous melatonin content and upregulated melatonin biosynthetic gene expression. Besides that, exogenous melatonin application enhanced the endogenous phytohormone contents such as salicylates, jasmonic acid associated with improving the tolerance to plant diseases. Moreover, exogenous melatonin application significantly alleviated the Huanglongbing damage via improved endogenous plant hormone contents as well as the transcript levels of their biosynthetic genes [29]. Moreover, exogenous melatonin application improved the endogenous melatonin level, upregulated the genes involved in the biosynthesis of melatonin and free radical defense, thus decreased CLas bacterial population and negatively regulated CLas-infection [32]. In cherry tomato, exogenous melatonin treatment can suppress the food-borne Bacillus proliferation such as *Bacillus cereus*, *Bacillus licheniformis*, and *Bacillus subtilis*. Further research showed that melatonin possessed the antibacterial activity against *B. subtilis* by inhibiting cell division, oxidative phosphorylation as well as reducing biofilm formation. In addition, melatonin can also improve the antioxidant capacity and induce phenolics and ethylene biosynthesis as well as upregulate the genes *PT16* and *PR1b1* involved in pathogenesis-related responses in cherry tomato [30]. Another interesting study showed that phytomelatonin can prevent bacterial invasion during nighttime. Phytomelatonin receptor 1 is involved in the process that phytomelatonin acts as a darkness signal role in circadian stomatal closure to withstand bacterial invasion at night [31]. In cowpea, glutathione peroxidase (GSH-PX), catalase (CAT), and salicylic acid (SA) levels were significantly upregulated, and hydrogen peroxide (H_2_O_2_) levels were significantly downregulated in melatonin-treated root samples [33]. These studies can provide a better understanding of melatonin’s defensive role response to deal with bacterial disease. With further functions of melatonin in response to bacterial stress uncovered in horticultural crops, the potential application of melatonin in horticultural crops to cope with bacterial stress will be possible.

## Fungal diseases

Fungal diseases pose a significant threat to plant growth, leading to substantial production and economic losses in horticultural crops worldwide [34]. Previous studies have demonstrated that melatonin plays a crucial role in enhancing the tolerance of horticultural crops to fungal stress [16–19, 22–25, 27, 28, 35–50]. For example, exogenous melatonin treatment has been reported to enhance tolerance to Marssonina apple blotch, which causes premature defoliation in apple trees. Further research revealed that melatonin pretreatment helps regulate intracellular H₂O₂ levels and boosts the activity of defense-related enzymes, thereby enhancing disease resistance [16]. *Fusarium oxysporum* f.sp. cubense (Foc), a widespread pathogen in major banana-growing regions, significantly reduces yields. Studies have shown that melatonin treatment enhances resistance to Foc by regulating HSP90 transcript levels [17]. In *Malus domestica*, melatonin treatment improves the rhizosphere environment and modifies the structure of the endophytic microbial community by reducing phloridzin levels in both rhizosphere soil and roots, thereby alleviating apple replant disease [47]. It also mitigates disease symptoms by promoting plant height, stem diameter, and leaf area, as well as increasing photosynthetic rate, CO₂ assimilation, and chlorophyll concentration. Further studies have shown that melatonin treatment upregulates the expression of genes associated with antioxidant and ROS-scavenging enzymes [35, 44]. Notably, melatonin treatment significantly increases the activities of 4-coumarate coenzyme A ligase, cinnamic acid 4-hydroxylase, and glucose phosphate isomerase in *Malus domestica* infected by *Penicillium expansum* [46]. In litchi, downy blight infection commonly occurs, severely affecting the quality of harvested fruits. However, melatonin application alleviated disease symptoms in litchi by activating phenylalanine ammonia-lyase, cinnamate-4-hydroxylase, and 4-hydroxycinnamate CoA ligase, along with increased accumulation of phenolic and flavonoid compounds. Additionally, melatonin treatment enhances the levels of nicotinamide adenine dinucleotide phosphate and the activities of glucose-6-phosphate dehydrogenase and 6-phosphogluconic acid dehydrogenase. Moreover, melatonin treatment increases the activities of H^+^-ATPase and Ca^2+^-ATPase. These findings suggest that melatonin enhances litchi resistance to downy blight by modulating the phenylpropanoid and pentose phosphate pathways, as well as energy metabolism [28]. In *Vitis vinifera*, melatonin treatment reduces disease incidence by promoting the biosynthesis and accumulation of total phenolics and flavonoids, decreasing malondialdehyde levels, limiting cell membrane permeability, and significantly enhancing the activities of SOD, POD, CAT, and PAL [37]. In pear, melatonin treatment significantly enhances disease resistance by inhibiting lesion development, increasing endogenous melatonin levels, reducing ROS accumulation, activating ROS-scavenging enzymes, maintaining the ascorbate-glutathione cycle in a reduced state, and upregulating autophagy-related gene expression [41]. Melatonin also works synergistically with jasmonic acid and phlorizin to enhance the antioxidant defense system and regulate the phenylpropanoid pathway in pear fruit, thus improving resistance to ring rot disease [48]. In mango, melatonin treatment significantly upregulates the activities of key enzymes in the phenylpropanoid pathway (e.g. PAL, C4H, 4CL) and pathogenesis-related (PR) proteins, along with increased accumulation of flavonoids, anthocyanins, lignin, and total phenolic compounds. Additionally, melatonin significantly reduces the activity of cell wall-degrading hydrolases (e.g. polygalacturonase, pectin methylesterase) and soluble pectin levels, delays cellulose and protopectin degradation, and enhances resistance to Colletotrichum gloeosporioides by stimulating defense enzyme activities (e.g. SOD, POD, CAT), promoting secondary metabolite biosynthesis, and inhibiting pectin degradation [49].

In vegetables, melatonin also plays a vital role in enhancing plant responses to fungal stress. For instance, cucumber downy mildew is a serious threat to yield. Melatonin treatment improves resistance by upregulating defense-related gene expression and enhancing antioxidant enzyme activity. Besides, melatonin application effectively decreased the relative electrolyte leakage as well as levels of MDA, thereby protecting membrane integrity. Moreover, melatonin treatment can also improve photosynthetic efficiency and nitrogen metabolism capacity [25]. Similarly, melatonin treatment enhances resistance to *Fusarium oxysporum* in cucumber by increasing arbuscular mycorrhizal (AM) colonization in roots. Subsequent analysis revealed that melatonin significantly increases net photosynthetic rate, stomatal conductance, intercellular CO₂ concentration, transpiration rate, and dry biomass under *Fusarium*-induced stress. Additionally, melatonin application reduces electrolyte leakage, MDA content, and H₂O₂ accumulation in cucumber plants infected with *Fusarium* [27]. In tomato, melatonin treatment suppresses gray mold development by inducing ROS accumulation, increasing endogenous melatonin and salicylic acid levels, and enhancing chitinase and β-1,3-glucanase activities. Additionally, melatonin modulates the phenylpropanoid pathway, inducing signaling molecules that contribute to enhancing fungal resistance during postharvest storage [22]. The treatment also activates calcium-dependent protein kinases and respiratory burst oxidase homologs involved in ROS accumulation, increases salicylic acid (SA) and lignin content, and upregulates SA pathway-related and defense genes such as *SlNPR1*, *SlPR1*, *SlPR2*, *SlGLU*, *SlTDC*, *SlSNAT*, and *SlASMT* [38, 39]. Furthermore, it inhibits cell death by scavenging reactive oxygen species, thereby preventing Botrytis cinerea from establishing infection sites [50]. Similarly, melatonin treatment significantly enhances resistance to *Botrytis cinerea* by activating defense-related enzymes and reducing H₂O₂ levels. In addition, melatonin increases methyl jasmonate levels, upregulates the expression of genes such as *SlLoxD*, *SlAOC*, and *SlPI II*, while downregulating *SlMYC2* and *SlJAZ1* [24]. In potato, melatonin treatment significantly enhances tolerance to late blight by inhibiting mycelial growth and modulating cellular ultrastructure [18]. In watermelon, melatonin treatment promotes endogenous melatonin biosynthesis, thereby increasing resistance to powdery mildew. Further research showed that melatonin application upregulates genes involved in pathogen-associated molecular pattern (PAMP)- and effector-triggered immunity (ETI)-related defenses [19]. However, some studies have reported adverse effects of melatonin application. In citrus, melatonin treatment was initially found beneficial in coping with *Penicillium digitatum* (Pd), a major postharvest pathogen. However, exogenous melatonin application did not inhibit Pd growth and instead significantly accelerated the manifestation of green mold symptoms. Moreover, melatonin reduced H₂O₂ levels, thereby weakening resistance to green mold by modulating ROS-scavenging defense mechanisms in citrus fruit [23]. In radish and pak choi, melatonin treatment promotes vigorous seedling growth and strengthens plant immunity by improving cellular organelle function, upregulating the biosynthesis of antioxidant enzymes, chitin, organic acids, and defense proteins, while simultaneously enhancing growth, increasing antioxidant activity, and boosting photosynthetic pigment accumulation [43, 45].

## Viral diseases

Plant virus diseases, seriously threatens the healthy development of horticultural crops, and resulting in huge losses in the world [51]. Unlike the diseases caused by bacteria and fungi, viral diseases are extremely hard to control [52]. Although few studies have reported on melatonin involvement in horticultural crops in response to the virus disease compared with research on bacterial and fungal diseases, there are still reports indicating that exogenous melatonin application can enhance resistance to viruses [21, 26, 53, 54]. For example, exogenous melatonin treatment can increase antiviral activity as well as gene expression, such as *PR1, PR5,* and significantly reduce relative levels of virus RNA. Besides, melatonin treatment can also increase salicylic acid and nitric oxide accumulations [26]. In apple, another study indicated that exogenous melatonin application significantly increased the number of shoots. Moreover, melatonin application increased the level of endogenous IAA and reduced the apple stem grooving virus concentration. Further research indicated that exogenous melatonin treatment can enlarge the virus-free area through virus localization [21]. In eggplants, melatonin and salicylic acid treatment can significantly increase chlorophyll content as well as the antioxidant enzyme activity to improve the resistance of eggplant to alfalfa mosaic virus infection. In *Cucumis sativus*, melatonin treatment can control the cucumber green mottle mosaic virus by upregulating the defense-related gene *CRISP1* [54]. In addition, exogenous melatonin and salicylic acid treatment significantly alleviated the oxidative damage by regulating the reduction of H_2_O_2_, O2-, OH-, and MDA [53]. These four studies provide a potential application for melatonin-mediated horticultural crops resistance to viruses.

**Table 1:** Effects of exogenous application of melatonin on pathogen-induced diseases in fruit crops.

Pathogen type	Pathogen name	Plant species	Effective dose of melatonin (μM)	Effect	Physiological and molecular mechanisms	References
Bacterium	*Candidatus Liberibacter asiaticus* (Huanglong bing)	*Citrus*	Leaf treatment (100 μM)	Enhancing disease resistance	Improved endogenous plant hormone levels and upregulated biosynthetic gene transcripts	[29]
Fungus	*Diplocarpon mali* (Apple blotch)	*Malus prunifolia*	Leaf treatment (50 μM)	Enhancing disease resistance	Maintained intracellular H_2_O_2_ and increased antioxidant enzyme activity	[16]
	*Fusarium oxysporum f.sp. cubense* (Fusarium wilt)	*Musa acuminata*	Seedling treatment (100 μM)	Enhancing disease resistance	Regulated JA, SA, IAA, and ET levels, improving resistance to Fusarium wilt	[17]
	*Penicillium digitatum* (Green mold)	*Citrus reticulata*	Fruit treatment (50 μM)	Reducing disease resistance	Decreased H_2_O_2_ and defense-related enzyme activity	[23]
	Fungal decay	*Fragaria anannasa*	Fruit treatment (100 μM)	Enhancing disease resistance	Increased H_2_O_2_ levels, reduced CAT and APX activity	[55]
	*Peronophythora litchii* (Downy blight)	*Litchi chinensis*	Fruit treatment (250 μM)	Enhancing disease resistance	Upregulated PAL, C4H, and 4CL; increased phenolics and flavonoids	[28]
	Apple replant disease	*Malus domestica*	Root treatment (200 μM)	Enhancing disease resistance	Improved growth parameters and photosynthesis	[35]
	Apple replant disease	*Malus domestica*	Root treatment (200 μM)	Enhancing disease resistance	Upregulated antioxidant and ROS-scavenging gene expression	[44]
	Apple replant disease	*Malus domestica*	Seedling treatment (200 μM)	Enhancing disease resistance	Improved rhizosphere conditions and microbiota composition	[47]
	*Penicillium expansum* (Postharvest rot)	*Malus domestica*	Fruit treatment (50 μM)	Enhancing disease resistance	Enhanced PPO, G6PDH, GPI activity; increased secondary metabolites	[46]
	*Botrytis cinerea* (Gray mold)	Grapevine	Fruit treatment (2 mM)	Reducing disease incidence	Stimulated phenolics, reduced MDA, stabilized membranes	[37]
	*Botryosphaeria dothidea* (Ring rot)	*Pyrus spp*. (Pear)	Fruit treatment (100 μM)	Enhancing disease resistance	Reduced lesion size, boosted melatonin, activated ROS-scavengers	[41]
	*Botryosphaeria dothidea* (Ring rot)	*Pyrus spp.* (Pear)	Fruit treatment (100 μM)	Enhancing disease resistance	Activated phenylpropanoid pathway and antioxidant enzymes	[48]
	*Colletotrichum gloeosporioides* (Anthracnose)	*Mangifera indica* (Mango)	Fruit treatment (0.2 μM)	Enhancing disease resistance	Enhanced defense enzymes and secondary metabolites	[49]
Virus	Apple stem grooving virus	*Malus domestica*	Medium supplementation (15 μM)	Enhancing disease resistance	Decreased virus concentration, increased IAA and shoot number	[21]

**Table 2:** Effects of exogenous application of melatonin on pathogen-induced diseases in vegetable crops.

Pathogen type	Pathogen name	Plant name	Effective dose of melatonin (μM)	Functions	Physiological and molecular mechanisms	References
Bacterium	*Xanthomonas axonopodis* (Cassava bacterial blight)	*Manihot esculenta* (Cassava)	Leaf treatment (200 μM)	Enhancing disease resistance	Upregulated melatonin biosynthesis genes and increased endogenous melatonin concentration	[20]
	Food-borne *Bacillus spp*.	*Solanum lycopersicum* (Cherry tomato)	Fruit treatment (10 mM)	Enhancing disease resistance	Improved antioxidant capacity and induced phenolics and ethylene biosynthesis	[30]
Fungus	*Pseudoperonospora cubensis* (Downy mildew)	*Cucumis sativus* (Cucumber)	Seedling treatment (100 μM)	Enhancing disease resistance	Increased antioxidant enzyme activity and gene expression; decreased MDA and electrolyte leakage	[25]
	Leaf blight	*Lilium spp.*	Root treatment (2 mM)	Enhancing disease resistance	Enriched defense-related DEGs in MAPK signaling, hormone signaling, and phenylpropanoid pathways	[42]
	Leaf blight	*Raphanus sativus* (Radish)	Seedling treatment (500 μM)	Significantly less blight	Enhanced organelle function and antioxidant enzyme biosynthesis, improved growth and immunity	[45]
	*Fusarium oxysporum*	*Cucumis sativus*	Seedling treatment (100 μM)	Enhancing disease resistance	Promoted arbuscular mycorrhizal colonization and reduced disease index	[27]
	*Podosphaera xanthii* (Powdery mildew)	*Citrullus lanatus*	Leaf treatment (1000 μM)	Enhancing disease resistance	Improved resistance to powdery mildew and Phytophthora capsici	[19]
	*Phytophthora infestans*	*Solanum tuberosum* (Potato)	Seedling treatment (10 μM)	Enhancing disease resistance	Suppressed mycelial growth, altered cell ultrastructure, and reduced pathogen stress tolerance	[18]
	*Botrytis cinerea* (Gray mold)	*Solanum lycopersicum*	Fruit treatment (50–100 μM)	Enhancing disease resistance	Elevated antioxidant enzyme activity, reduced H_2_O_2_, increased methyl jasmonate and SA levels, and upregulated defense genes (e.g. *SlNPR1*, *SlPR1*, *SlPR2*, *SlGLU*)	[22, 24, 38, 39, 50]
	*Plasmodiophora brassicae* (Clubroot)	*Brassica rapa ssp. chinensis* (Pak choi)	Leaf treatment (50 mM)	Enhancing disease resistance	Improved growth and antioxidant activity, increased chlorophyll and carotenoid levels	[43]
Virus	Tobacco mosaic virus	*Solanum lycopersicum*	Seedling treatment (100 μM)	Enhancing disease resistance	Increased salicylic acid and nitric oxide accumulation	[26]
	Alfalfa mosaic virus	*Solanum melongena*	Leaf treatment (100 μM)	Enhancing disease resistance	Increased chlorophyll, carotenoids, antioxidant enzymes, and related gene expression	[53]
	Cucumber green mottle mosaic virus	*Cucumis sativus*	Root irrigation treatment (50 μM)	Control CGMMV infection	Upregulated *CRISP1* gene involved in virus defense	[54]
Mixed	Cowpea wilt	*Cowpea*	Seedling treatment (100 μM)	Enhancing disease resistance	Upregulated GSH-PX, CAT, SA; downregulated H₂O₂ levels	[33]

**Figure 1:**
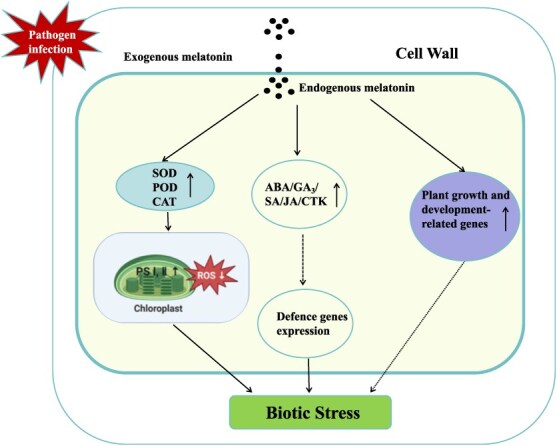
The proposed model of melatonin-induced disease resistance.

## Other diseases

Other stresses such as parasitic nematodes, weeds, and insects can also affect the horticultural crops growth and production. However, no sufficient research indicates that melatonin has a positive influence on parasitic nematodes, weeds, and insects or that it is involved in the response process to these stresses, so further study in this area is needed.

Melatonin alleviates pathogen-induced diseases in horticultural crops via physiological and molecular processes.

In fruit crops, melatonin treatment can keep up H_2_O_2_ concentrations, enhanced antioxidant enzyme activity, improve height, stem diameter, leaf area, and the dry weights of roots, leaves, and stems as well as improve enzyme activities, acid contents to defense against the disease in *Malus prunifolia* [16, 35, 44, 46], a similar process occurs in *Citrus reticulata* to alleviate *Penicillium digitatum* [23], in pear [41], in grapevine [37], in Cherry tomato [38, 39], in Radish [45]. In *Fragaria anannasa*, exogenous melatonin treatment can improve the fungal decay resistance by increasing H_2_O_2_ concentrations [55]. However, in *Litchi chinensis*, it can enhance the activities of phenylalanine ammonia-lyase, cinnamate-4-hydroxylase as well as 4-hydroxycinnamate CoA ligase while promoting the accumulations of phenolics and flavonoids to alleviate the *Peronophythora litchii* [28]. Melatonin treatment can regulate of JA, SA, IAA, and ET, thus improving banana tolerance to *Fusarium wilt* in *Musa acuminata* [17], and also enhance the endogenous plant hormone contents as well as their biosynthetic genes transcript levels involving in stress to alleviate the Huanglongbing in *Citrus* [29]. In *Malus domestica*, melatonin treatment can improve in height, stem diameter, leaf area, and the dry weights of roots, leaves, and stems, and increased the photosynthetic rate as well as higher CO_2_ assimilation rates and chlorophyll levels to manage the apple replant disease [34], and decrease virus concentration to deal with apple stem grooving virus [21]. In vegetable crops, melatonin application can improve the antioxidant capacity and induce phenolics and ethylene biosynthesis to improve the resistance to deal with *food-borne Bacillus* in *Cherry tomatoes* [30], and induce a ROS accumulation, increase endogenous melatonin and SA concentration as well as enhance activities of chitinase and β-1,3-glucanase to defense the gray mold in tomato [22], and increase the activities of defense-related enzymes and decrease hydrogen peroxide content, enhance antioxidant enzyme activities and increase methyl jasmonate content to handle the *Botrytis cinerea* in *Solanum lycopersicum* [24], the similar process to defense the Tobacco mosaic virus in *Solanum lycopersicum* [26]. In potato, melatonin treatment can significantly attenuated the potato late blight by inhibiting mycelial growth, changing cell ultrastructure, and reducing stress tolerance of *P. infestans* [18], and also significantly increased in the morphological criteria, chlorophyll and carotenoid content, antioxidant enzymes, and gene expression of some enzymes to defend against the Alfalfa mosaic virus in *Solanum melongena* [53]. Overall, melatonin alleviates pathogen-induced diseases in horticultural crops by adjusting the enzyme activity, related gene expression of plant hormone involved in the process to handle the biotic stress. Previous research demonstrates in Table 1 that melatonin has a significant influence in fruit crops to cope with biotic stresses, and Table 2 shows that melatonin also plays a key role in vegetable crops.

## Conclusions and perspectives

Many studies have shown that melatonin is involved in the process by which horticultural crops cope with various biotic stress. In this review, we systematically summarized the melatonin-mediated responses in horticultural crops involved in biotic stress tolerance, with an emphasis on the melatonin biosynthesis and metabolic pathways and several major biotic stresses such as bacteria, fungi, virus stress (Fig. 1). The major antioxidant processes mediated by melatonin in horticultural crops under biotic stress induce triggering a defense response, improve antioxidant enzyme activity, such as SOD, POD, CAT, and reduce ROS content, protect chloroplast structure, thereby preventing damage under biotic stress in horticultural crops. Besides, melatonin can regulate the gene expression involved in plant growth and development as well as stress response. There are limited transcriptomic, metabolome, and proteome studies on melatonin-treated horticultural crops. Hence, we suggest that omics analysis should be given priority in the process of melatonin involvement in coping with biotic stress in horticultural plants, including the regulation of melatonin biosynthesis and its interactions with other factors at the transcriptional, metabolome or proteome. More omics studies are necessary to identify key genes, enhancing the tolerant for horticultural crops cultivars. Moreover, research showed that melatonin can regulate the gene expression of abscisic acid, cytokinin, gibberellin, salicylic acid, and jasmonic acid under biotic stress. These plant hormones play an important role in horticultural crops to cope with biotic stress. Therefore, cross-talk between exogenous melatonin application and other plant hormone should be an important focus of research to improve the tolerance under biotic stress in horticultural crops. Up to now, few studies have reported that melatonin is involved in the defense process to viruses and other biotic stresses such as parasitic nematodes, weeds, and insects. Therefore, future studies should focus particularly on viral pathogens. Although some studies have reported negative effect studies of melatonin on biotic stress in horticultural crops, current research in this area is limited. The potential negative effects of melatonin treatment on horticultural crops should be evaluated, particularly in field experiments. When the security of exogenous melatonin application is fully evaluated, the application of melatonin in horticultural crops can be scaled up for commercial and agricultural use. In conclusion, melatonin is now widely used in the research of horticultural crops, and more and more functions have been identified. With rigorous safety evaluations completed, melatonin is expected to become a natural, effective, and economical growth regulator for horticultural crops, thereby being widely applied in horticultural production.

The figure displays a melatonin-modulated feedback system in plants under biotic stress. Biotic stressors cause an accumulation of SOD, POD, CAT and ABA, GA_3_, SA, JA, and CTK, stimulating the production of endogenous melatonin. Plant melatonin can remove ROS and upregulate plant growth-, development-, and defense-related gene expression.

## References

[1] Singh Y, Prajapati S. Status of horticulture crops: identifying the need for transgenic traits. In: Rout GR, Peter KV, eds. Genetic. Engineering of Horticultural Crops. Elsevier: Jabalpur, India, 2018,1–21

[2] Gao T, Liu X, Tan K. et al. Introducing melatonin to the horticulture industry:physiological roles, potential applications, and challenges. Hortic Res. 2022;9:uhac094 35873728 10.1093/hr/uhac094 PMC9297156

[3] Tiwari A, Sharma P, Khan M. et al. Medicinal plants-international journal of Phytomedicines and related industries. Med Plants Int J Phytomed. 2009;2:117–23

[4] Zadoks J . The potato murrain on the European continent and the revolutions of 1848. Potato Res. 2008;51:5–45

[5] Massart S, Jijakli H, Kummert J. Apple stem grooving virus. In: Hadidi A, Barba M, Candresse T. et al., eds. Virus and Virus like Diseases of Pome and Stone Fruits. APS Press, 2011,29–33

[6] Mannini F, Digiaro M. The effects of viruses and viral diseases on grapes and wine. In: Meng B, Martelli G, Golino D. et al., eds. Grapevine Viruses: Molecular Biology, Diagnostics and Management. Springer: Cham, 2017,453–82

[7] Martelli. An overview on grapevine viruses, viroids, and the diseases they cause. In: Meng B, Martelli G, Golino D, Fuchs M, eds. Grapevine Viruses: Molecular Biology, Diagnostics and Management. Springer International Publishing: Cham, 2017,31–46

[8] Hao X, Jiao B, Wang M. et al. In vitro biological indexing of grapevine leafroll-associated virus 3 in red-and white-berried grapevines (*Vitis vinifera*). Aust J Grape Wine Res. 2021;27:483–90

[9] Wang M, Bi W, Bettoni J. et al. Shoot tip cryotherapy for plant pathogen eradication. Plant Pathol. 2022;71:1241–54

[10] Lerner A, Case J, Takahashi Y. et al. Isolation of melatonin, the pineal gland factor that lightens melanocytes. J Am Chem Soc. 1958;80:2587–630

[11] Dubbels R, Reiter RJ, Klenke E. et al. Melatonin in edible plants identified by radioimmunoassay and by high performance liquid chromatography-mass spectrometry. J Pineal Res. 1995;18:28–31 7776176 10.1111/j.1600-079x.1995.tb00136.x

[12] Hattori A, Migitaka H, Iigo M. et al. Identification of melatonin in plants and its effects on plasma melatonin levels and binding to melatonin receptors in vertebrates. Biochem Mol Biol Int. 1995;35:627–34 7773197

[13] Liang D, Ni Z, Xia H. et al. Exogenous melatonin promotes biomass accumulation and photosynthesis of kiwifruit seedlings under drought stress. Sci Hortic. 2019;246:34–43

[14] Wang T, Hu M, Yuan D. et al. Melatonin alleviates pericarp browning in litchi fruit by regulating membrane lipid and energy metabolisms. Postharvest Biol Technol. 2020;160:111–066

[15] Li H, Guo Y, Lan Z. et al. Methyl jasmonate mediates melatonin-induced cold tolerance of grafted watermelon plants. Hortic Res. 2021;8:57 33750773 10.1038/s41438-021-00496-0 PMC7943586

[16] Yin H, Wang P, Li M. et al. Exogenous melatonin improves Malus resistance to Marssonina apple blotch. J Pineal Res. 2013;54:426–34 23356947 10.1111/jpi.12038

[17] Wei Y, Hu W, Wang Q. et al. Identification, transcriptional and functional analysis of heat-shock protein 90s in banana (*Musa acuminata* L.) highlight their novel role in melatonin-mediated plant response to Fusarium wilt. J Pineal Res. 2017;62:62 10.1111/jpi.12367 27627033

[18] Zhang S, Zheng X, Reiter R. et al. Melatonin attenuates potato late blight by disrupting cell growth, stress tolerance, fungicide susceptibility and homeostasis of gene expression in *Phytophthora infestans*. Front Plant Sci. 2017;8:1993 29209352 10.3389/fpls.2017.01993 PMC5702310

[19] Mandal M, Suren H, Ward B. et al. Differential roles of melatonin in plant-host resistance and pathogen suppression in cucurbits. J Pineal Res. 2018;65:e12505 29766569 10.1111/jpi.12505

[20] Wei Y, Chang Y, Zeng H. et al. RAV transcription factors are essential for disease resistance against cassava bacterial blight via activation of melatonin biosynthesis genes. J Pineal Res. 2018;64:64 10.1111/jpi.12454 29151275

[21] Chen L, Wang M, Li J. et al. Exogenous application of melatonin improves eradication of apple stem grooving virus (ASGV) from the infected in vitro shoots by shoot tip culture. Plant Pathol. 2019;68:997–1006

[22] Li S, Xu Y, Bi Y. et al. Melatonin treatment inhibits gray mold and induces disease resistance in cherry tomato fruit during postharvest. Postharvest Biol Technol. 2019;157:110962

[23] Lin Y, Fan L, Xia X. et al. Melatonin decreases resistance to postharvest green mold on citrus fruit by scavenging defense-related reactive oxygen species. Postharvest Biol Technol. 2019;153:21–30

[24] Liu C, Chen L, Zhao R. et al. Melatonin induces disease resistance to Botrytis cinerea in tomato fruit by activating jasmonic acid signaling pathway. J Agric Food Chem. 2019;67:6116–24 31084000 10.1021/acs.jafc.9b00058

[25] Sun Y, Liu Z, Lan G. et al. Effect of exogenous melatonin on resistance of cucumber to downy mildew. Sci Hortic. 2019;255:231–41

[26] Zhao L, Chen L, Gun P. et al. Exogenous application of melatonin improves plant resistance to virus infection. Plant Pathol. 2019;68:1287–95

[27] Ahammed G, Mao Q, Yan Y. et al. Role of melatonin in arbuscular mycorrhizal fungi-induced resistance to fusarium wilt in cucumber. Phytopathology. 2020;110:999–1009 32096697 10.1094/PHYTO-11-19-0435-R

[28] Zhang Z, Wang T, Liu G. et al. Inhibition of downy blight and enhancement of resistance in litchi fruit by postharvest application of melatonin. Food Chem. 2021;347:129009 33444889 10.1016/j.foodchem.2021.129009

[29] Nehela Y, Killiny N. Melatonin is involved in citrus response to the pathogen Huanglongbing via modulation of phytohormonal biosynthesis. Plant Physiol. 2020;184:2216–39 32843523 10.1104/pp.20.00393 PMC7723116

[30] Zhu G, Sha P, Zhu X. et al. Application of melatonin for the control of food-borne bacillus species in tomatoes. Postharvest Biol Technol. 2021;181:111656

[31] Li X, Rengel Z, Chen Q. Phytomelatonin prevents bacterial invasion during nighttime. Trends Plant Sci. 2022;27:331–4 34996703 10.1016/j.tplants.2021.12.008

[32] Nehela Y, Killiny N. Infection with phytopathogenic bacterium inhibits melatonin biosynthesis, decreases longevity of its vector, and suppresses the free radical-defense. J Pineal Res. 2018;65:12511 10.1111/jpi.12511 29786865

[33] Gan Y, Tu Z, Yang Y. et al. Enhancing cowpea wilt resistance: insights from gene coexpression network analysis with exogenous melatonin treatment. BMC Plant Biol. 2024;24:1–14 38918732 10.1186/s12870-024-05289-w PMC11197195

[34] Meno L, Abuley I, Escuredo O. et al. Factors influencing the airborne sporangia concentration of Phytophthora infestans and its relationship with potato disease severity. Sci Hortic. 2023;307:111520

[35] Li C, Zhao Q, Gao T. et al. The mitigation effects of exogenous melatonin on replant disease in apple. J Pineal Res. 2018;65:65 10.1111/jpi.12523 30230015

[36] Li T, Wu Q, Zhu H. et al. Comparative transcriptomic and metabolic analysis reveals the effect of melatonin on delaying anthracnose incidence upon postharvest banana fruit peel. BMC Plant Biol. 2019a;19:289 31262259 10.1186/s12870-019-1855-2 PMC6604187

[37] Li Z, Zhang S, Xue J. et al. Exogenous melatonin treatment induces disease resistance against Botrytis cinerea on post-harvest grapes by activating Defence responses. Foods. 2022;11:2231 35953999 10.3390/foods11152231 PMC9367934

[38] Li S, Cheng Y, Yan R. et al. Preharvest spray with melatonin improves postharvest disease resistance in cherry tomato fruit. Postharvest Biol Technol. 2022a;193:112055

[39] Li S, Huan C, Liu Y. et al. Melatonin induces improved protection against Botrytis cinerea in cherry tomato fruit by activating salicylic acid signaling pathway. Sci Hortic. 2022b;304:111299

[40] Thangaraj K, Liu S, Li J. et al. Exogenous melatonin alleviates sooty mould on tea plants (*Camellia sinensis* L.). Sci Hortic. 2022;299:111056

[41] Wang Y, Wang G, Xu W. et al. Exogenous melatonin improves pear resistance to Botryosphaeria dothidea by increasing Autophagic activity and sugar/organic acid levels. Phytopathology. 2022;112:1335–44 34989595 10.1094/PHYTO-11-21-0489-R

[42] Xie X, Han Y, Yuan X. et al. Transcriptome analysis reveals that exogenous melatonin confers Lilium disease resistance to Botrytis elliptica. Front Genet. 2022;13:892674 35774503 10.3389/fgene.2022.892674 PMC9237519

[43] Gulzar S, Manzoor M, Liaquat F. et al. Effects of melatonin and Trichoderma harzianum on pak choi yield, chlorophyll contents and antioxidant defense system under clubroot disease. S Afr J Bot. 2023;158:292–300

[44] He X, Yin B, Zhang J. et al. Exogenous melatonin alleviates apple replant disease by regulating rhizosphere soil microbial community structure and nitrogen metabolism. Sci Total Environ. 2023;884:163830 37137374 10.1016/j.scitotenv.2023.163830

[45] Li J, Huang T, Xia M. et al. Exogenous melatonin mediates radish (*Raphanus sativus*) andAlternaria brassicae interactionin a dose-dependent manner. Front Plant Sci. 2023;14:1126669 36923135 10.3389/fpls.2023.1126669 PMC10009256

[46] Wang M, Li Y, Li C. et al. Melatonin induces resistance against Penicillium expansum in apple fruit through enhancing phenylpropanoid metabolism. Physiol Mol Plant Pathol. 2023;127:102082

[47] Ma C, Du P, Cao B. et al. Melatonin alleviates apple replant disease by regulating the endophytic microbiome of roots and phloridzin accumulation. Microbiol Res. 2024;289:14 10.1016/j.micres.2024.127897 39243684

[48] Xu H, Zhang S, Liang C. et al. Melatonin enhances resistance to Botryosphaeria dothidea in pear by promoting jasmonic acid and phlorizin biosynthesis. BMC Plant Biol. 2024;24:1–14 38811892 10.1186/s12870-024-05187-1 PMC11134937

[49] Lu D, Ren Y, Yan T. et al. Melatonin improves the postharvest anthracnose resistance of mango fruit by regulating antioxidant activity, the phenylpropane pathway and cell wall metabolism. Eur J Plant Pathol. 2025;171:17–36

[50] Shan Q, Zhao D, Cao B. et al. Jasmonic acid and nitric oxide orchestrate a hierarchical melatonin cascade for Botrytis cinerea resistance in tomato. Plant Physiol. 2025;197:kiaf078 39977124 10.1093/plphys/kiaf078

[51] Devendran R, Kumar M, Ghosh D. et al. Capsicum-infecting begomoviruses as global pathogens: host-virus interplay, pathogenesis, and management. Trends Microbiol. 2022;30:170–84 34215487 10.1016/j.tim.2021.05.007

[52] Hadidi A, Barba M. Economic impact of pome and stone fruit viruses and viroids. In: Hadidi A, Barba M, Candresse W, Jelkman W, eds. Virus and Virus-like Diseases of Pome and Stone Fruits. The American Phytopathological Society: St Paul, MN, USA, 2011,1–7

[53] Sofy A, Sofy M, Hmed A. et al. Molecular characterization of the alfalfa mosaic virus infecting Solanum melongena in Egypt and the control of its deleterious effects with melatonin and salicylic acid. Plants. 2021;10:459 33670990 10.3390/plants10030459 PMC7997183

[54] Yang L, Li Q, Han X. et al. A cysteine-rich secretory protein involves in phytohormone melatonin mediated plant resistance to CGMMV. BMC Plant Biol. 2023;23:215 37098482 10.1186/s12870-023-04226-7 PMC10127030

[55] Aghdam M, Fard J. Melatonin treatment attenuates postharvest decay and maintains nutritional quality of strawberry fruits (*Fragaria anannasa* cv. Selva) by enhancing GABA shunt activity. Food Chem. 2017;221:1650–7 27979142 10.1016/j.foodchem.2016.10.123

